# Identification and role of *CmLhcb2.1* in regulating low-light stress resistance in Chinese chestnut (*Castanea mollissima*)

**DOI:** 10.3389/fpls.2025.1552618

**Published:** 2025-03-10

**Authors:** Yong Yang, Xuan Wang, Jing Liu, Meng Wang, Liyang Yu, Dongsheng Wang, Jingshi Li, Yi Lu, Jingzheng Zhang, Haie Zhang

**Affiliations:** ^1^ College of Horticulture Science and Technology, Hebei Normal University of Science and Technology, Changli, Hebei, China; ^2^ Engineering Research Center of Chestnut Industry Technology, Ministry of Education, Hebei Normal University of Science and Technology, Qinhuangdao, Hebei, China; ^3^ Hebei Key Laboratory of Horticultural Germplasm Excavation and Innovative Utilization, College of Horticulture Science and Technology, Hebei Normal University of Science and Technology, Changli, Hebei, China

**Keywords:** Chinese chestnut, low light stress, RNA-seq, Yeast one-hybrid, Dual luciferase reporter assay, *CmLhcb2.1*, *CmGLK*

## Abstract

Chinese chestnut (*Castanea mollissima*) is a significant woody food plant that has garnered increasing attention due to its potential role in addressing food security challenges. However, low yield remains a critical issue facing the Chinese chestnut industry. One contributing factor to this low yield is insufficient light, particularly since Chinese chestnuts predominantly grow in mountainous regions. Therefore, the present study aims to investigate the intrinsic mechanisms underlying chestnut resistance to light stress, identify and validate genes associated with low light stress tolerance, and provide a foundation for targeted breeding of chestnut varieties that can withstand light stress. Studies have demonstrated that the light-harvesting chlorophyll a/b (*Lhca/b*) proteins play key roles in regulating the adaptation of plants to low-light stress. However, there have been no reports on the role of the *Lhca/b* gene family in the chestnut under light stress. We initially identified 17 *CmLhca/b* gene members across the chestnut genome and constructed a phylogenetic tree that divided them into five subgroups: the Lhca, the Lhcb, the CP24, the CP26, and the CP29 groups. CmLhcb2.1 and CmLhcb2.2 were grouped on the same branch with GhLhcb2.3 of upland cotton that involved in chlorophyll synthesis.The chestnut leaves exhibited phenotypic and transcriptomic differences under low and normal light conditions. By the 10th day of shading treatment, the leaves showed signs of damage, with the extent of damage intensifying as shading intensity increased. Additionally, the leaf color darkened due to the gradual increase in chlorophyll b content, which was correlated with increased shading intensity. The gene *CmLhcb2.1* was upregulated across all shading intensities. Specifically, quantitative reverse transcription PCR (qRT-PCR) confirmed the upregulation of *CmLhcb2.1* in chestnut under low-light stress. Overexpression studies in tobacco indicated that *CmLhcb2.1* enhances chestnut resistance to low-light stress by promoting chlorophyll b synthesis. Finally, yeast one-hybrid and dual-luciferase reporter assays confirmed that the transcription factor *CmGLK* positively regulated *CmLhcb2.1*. These findings lay a theoretical foundation for exploring how *CmLhcb2.1* regulates chestnut resistance to low-light stress.

## 1 Introduction

Chinese chestnut (*Castanea mollissima*), belonging to the *Fagaceae* family, is increasingly recognized as a woody food crop. However, low yield is a key issue that is challenging the Chinese chestnut industry. Photosynthesis is the foundation for morphological development and yield in the chestnut. Chestnuts are mostly found in the mountainous regions, especially the shaded slopes that receive insufficient sunlight. Therefore, insufficient light is one of the reasons for the low yield of Chinese chestnuts. Insufficient sunlight reduces the photosynthetic capacity, ultimately affecting chestnut yield (Chen et al., 2020; Dai et al., 2009; Wu et al., 2004).

Plants have evolved various strategies to improve photosynthetic efficiency and withstand low light (Wang et al., 2022). Plants such as rice and olive trees cope with low-light stress typically by increasing the leaf chlorophyll content (Alridiwirsah et al., 2018), reducing the chlorophyll a/b ratio (Wang et al., 2022), and adjusting leaf area and orientation (Ajmi et al., 2018; Liu et al., 2016). In addition to these physiological adjustments, plants also regulate the photosynthetic genes, including the light-harvesting chlorophyll a/b binding (Lhca/b) gene family, to adapt to low light conditions (Wang et al., 2022).

The chlorophyll a/b binding domain is a specific domain within the chlorophyll a/b binding proteins (LHC proteins) that is responsible for binding pigments such as chlorophyll a and chlorophyll b. This domain plays a crucial role in photosynthesis because it can stably bind these pigments, thereby efficiently capturing and transferring light energy. LHC proteins are the key components in converting light energy to chemical energy during photosynthesis (Lan et al., 2022). LHC proteins are found abundantly in the thylakoid membranes of chloroplasts, and they are associated with pigments that capture and transfer light (Green and Durnford, 1996). The LHC family includes Lhca/b, LIL (light-harvesting-like), PsbS (photosystem II subunit S), and FCII (ferrochelatase II) subfamilies (Klimmek et al., 2006; Zou and Yang, 2019). The Lhca/b proteins are further divided into Lhca and Lhcb proteins (Green et al., 1991; Jansson, 1999). Among these, the Lhca proteins are involved in the formation of the light-harvesting complex I (LHCI) in photosystem I (PSI), while Lhcb proteins participate in the formation of the light-harvesting complex II (LHCII) in photosystem II (PSII) (Waters et al., 2009). The Lhcb genes are further classified into two types. The first type encodes Lhcb1, Lhcb2, and Lhcb3 proteins, which assemble into a trimeric structure post-translationally and bind with the pigments, while the second type encodes the monomeric Lhcb4, Lhcb5, and Lhcb6 proteins, which independently bind with pigments to form specific complexes, namely CP29, CP26, and CP24 (Neilson and Durnford, 2010; Pietrzykowska et al., 2014).

With the continuous development of sequencing technologies and bioinformatic approaches, the *Lhca/b* family has been identified in several plant species. For instance, 57 members of the *Lhca/b* gene family (*BnLhca/b*) have been identified in *Brassica napus* (Xue et al., 2024), 28 (*PbrLhca/b*) in pear (Chen et al., 2020), 19 (*PpLhca/b*) in peach (Wang et al., 2023), and 27 (*MdLhca/b*) in apple (Zhao et al., 2020a). Moreover, studies have indicated the role of the *Lhca/b* gene family in regulating plant response to abiotic stress (Wang et al., 2023). In *Arabidopsis thaliana*, *AtLhcb1-6* regulates homeostasis of reactive oxygen species (ROS) and influences stomatal response to abscisic acid signals. Silencing *AtLhcb1-6* expression decreased Arabidopsis drought resistance (Xu et al., 2012). Consistent with this observation, overexpression of *LeLhcb2* significantly enhanced tobacco tolerance to cold stress (Deng et al., 2014), and *MdLhcb4.3* overexpression improved resistance of apple callus to osmotic and drought stresses (Zhao et al., 2020a). The aforementioned studies indicate that the Lhca/b gene family plays a crucial role in regulating plant responses to abiotic stress.

Numerous researchers have investigated plant Lhca/b genes in response to light stress. In *Juglans mandshurica* under low-light stress, the expression of *JmLhca2* and *JmLhca5* was significantly downregulated, while the expression of *JmLhcb1*, *JmLhca2*, *JmLhca3*, and *JmLhca5* was upregulated considerably (Zhang et al., 2022). In *Pinus koraiensis*, *PkLhca1* expression was significantly upregulated with a decrease in photosynthetically active radiation (*PAR*), while *PkLhcb7* expression was downregulated considerably (Li et al., 2022). Similarly, in *Quercus mongolica*, the expression of a few *QmLhca/b* subfamily members was significantly upregulated (Li et al., 2024).

Besides, studies have proven that the *Lhcb* genes underwent amplification during the evolutionary process to adapt to low-light stress. For example, Ma et al. found that compared with the freshwater plants near the water surface, seagrasses in the water have specifically amplified the *Lhcb* genes to enhance their light-harvesting capability (Ma et al., 2023). Interestingly, researches have proven the role of *Lhcb* genes in regulating chlorophyll content in plants. In upland cotton, silencing *GhLhcb2.3* reduced chlorophyll content (Zhang et al., 2021). Similarly, in *A. thaliana*, silencing *AtLhcb1* resulted in smaller leaves and reduced chlorophyll content (Lan et al., 2022). Consistent with these observations, introducing AcLhcb3.1/3.2 from kiwifruit into tobacco increased chlorophyll content (Luo et al., 2022). In *A. thaliana*, overexpression of tea *CsLhcII* restored leaf greenness (Chen et al., 2022). These findings collectively demonstrated the pivotal role of *Lhcb* genes in enhancing photosynthetic efficiency and chlorophyll regulation, thereby facilitating plant adaptation to varying light conditions.

In summary, there has been extensive research on the *Lhca/b* gene family, however, there are no studies on these genes in Chinese chestnut. Moreover, whether the *CmLhca/b* genes influence chestnut adaptation to low-light stress is unknown.

Therefore, the present study aimed to identify and characterize the *CmLhca/b* gene family members of the ‘N11-1’ chestnut (Wang et al., 2020). We analyzed the phylogenetic relationships, physicochemical properties, gene structure, and gene duplication types within the *CmLhca/b* gene family and the phenotypic and transcriptomic differences between chestnut leaves under low and normal light conditions. Further, *CmLhcb2.1* was overexpressed in tobacco to investigate its role in regulating the tolerance of chestnut to low-light stress. Finally, yeast one-hybrid and dual-luciferase reporter assays were performed to assess the regulatory role of the transcription factor on the gene *CmLhcb2.1*. The study’s findings will provide novel insights into the molecular mechanisms of chestnut adaptation to low-light stress.

## 2 Materials and methods

### 2.1 Identification of *CmLhca/b* genes and generation of a phylogenetic tree

The genomic data file (cm_n11_genome.fa) and the annotation file (cm_n11_gene_model.gff) of the ‘N11-1’ chestnut variety were downloaded from the China National Genomics Data Center (https://ngdc.cncb.ac.cn/gwh), while the Chlorophyll a/b binding domain file (ID PF00504) was obtained from the Pfam database (http://pfam-legacy.xfam.org/). Using the HMMER3.0 software, we conducted a comparative analysis to search for *CmLhca/b* potential genes across the entire chestnut genome (Potter et al., 2018). Further, the potential CmLhca/b protein sequences were uploaded to the InterProScan database (https://www.ebi.ac.uk/interpro/result/interprosca/), which includes protein domain annotation or functions from multiple databases such as Pfam, CDD (Conserved Domain Database), SMART (Simple Modular Architecture Research Tool), and PROSITE database. This approach allowed us to identify the true members of the *CmLhca/b* gene family of chestnut. We then aligned the protein sequences of the identified *CmLhca/b* members with the *Lhca/b* protein sequences reported in other plants, including *Gossypium hirsutum* (cotton), *Actinidia deliciosa* (kiwifruit), *Camellia sinensis* (tea), *Arabidopsis thaliana* (thale cress), *Ricinus communis* (castor bean), and *Jatropha curcas* (purging nut), and constructed a phylogenetic tree (**Supplementary Table S1**) to understand both their evolutionary relationship and classification (Chen et al., 2022; Luo et al., 2022; Zhang et al., 2021; Zhao et al., 2020b). The protein sequences were aligned using the MAFFT software, and the phylogenetic tree was constructed using the FastTree software following the maximum likelihood method using the default parameters (Poon et al., 2010; Rozewicki et al., 2019). Finally, the phylogenetic tree was visualized using the Chiplot online tool (https://www.chiplot.online/) (Xie et al., 2023).

### 2.2 Chromosome localization and physicochemical property analysis

The localization of the *CmLhca/b* gene family members on the chestnut chromosome was analyzed and visualized using the Gene Location Visualize (Advanced) module in the TBtools software (version 2.136) (Chen et al., 2023a). Then, the key physicochemical properties, such as amino acid count, molecular weight, isoelectric point, and instability index, of the CmLhca/b proteins were analyzed using the ExPASy ProtParam online tool (https://web.expasy.org/protparam/). The specific subcellular location of the member proteins was predicted using the BUSCA online tool (https://busca.biocomp.unibo.it/).

### 2.3 Analysis of gene structure, motif, and *cis*-acting elements

The motifs in the sequences of the CmLhca/b proteins were analyzed using the MEME online tool (https://meme-suite.org/). The conserved motifs were then uploaded to the InterProScan database (https://www.ebi.ac.uk/interpro/result/interprosca/) for functional annotation. The structure of the *CmLhca/b* genes was analyzed using the Gene Structure View (Advanced) module in the TBtools software. Finally, the motifs, the conserved domains, and the gene structures were visualized using TBtools software (Chen et al., 2023a). Further, using the Gtf/Gff3 Sequences Extract module in TBtools, the 2000 bp promoter sequences upstream of the ATG of the *CmLhca/b* genes were extracted. These sequences were uploaded to the PlantCARE database (http://bioinformatics.psb.ugent.be/webtools/plantcare/) to predict the *cis*-acting elements, and the quantities of each type of element on the different member proteins were analyzed (Chen et al., 2023a).

### 2.4 Analysis of collinearity of the *CmLhca/b* genes within the chestnut genome and with the genes of other species

The genomic and annotation files of oak, grape, and maize were downloaded from the China National Genomics Data Center and those of Arabidopsis and rice from the Ensembl Plants database (https://plants.ensembl.org/). The genomic and annotation files of the Japanese chestnut were downloaded from the Plant Genome Portal database (https://plantgarden.jp/en/index) and of the American chestnut from the Phytozome 13 database (https://phytozome-next.jgi.doe.gov/info/cdentata_v1_1.). Then, the MCScanX software was used to analyze the collinearity among the *CmLhca/b* genes within the chestnut genome and the collinearity of the *CmLhca/b* genes with the members in other species, including rice, maize, Arabidopsis, grape, oak, Japanese chestnut, and American chestnut (Wang et al., 2012). Finally, the Advanced Circos module in TBtools drew the collinearity map (Chen et al., 2023a).

Further, the duplication types of the *CmLhca/b* genes were determined using the DupGenfinder program (Qiao et al., 2019) and categorized into five types: Whole Genome Duplication (WGD), Transposed Duplication (TRD), Dispersed Duplication (DSD), Tandem Duplication (TD), and Proximal Duplication (PD) types. Then, the nonsynonymous substitution rate to the synonymous substitution rate (Ka/Ks) was calculated for the different gene pairs using the Simple Ka/Ks Calculator (NG) module in TBtools in order to assess evolutionary pressure and determine whether adaptive evolution has occurred (Chen et al., 2023a).

### 2.5 Treatment of plant materials and measurement of chlorophyll content

Two-year-old seedlings of the chestnut cultivar *Castanea mollissima* cv. ‘Yanbao’, exhibiting consistent growth potential and managed under normal water and fertilizer conditions, were used as experimental materials to investigate the differences in phenotypes, transcriptomes, and expression levels of chestnut leaves under varying shading intensities. The hybrid seedlings of ‘Yanbao’ and *Castanea mollissima* cv. ‘Funing_16’ served as the rootstocks. These seedlings were subjected to shading treatment using black shading nets, four shading intensities treatments (0%, 50%, 75%, and 95%) were adopted in this experiment. Each treatment was replicated three times, with three chestnut seedlings per replicate. Then, to ensure the stability of the shading treatments, the photosynthetically active radiation (*PAR*) was measured from May 24^th^ to May 26^th^ at 9:00 AM using the LI-600 Fluorescence Stomatal Measurement Instrument (LI-COR, Nebraska, USA) under different the shading treatments (**Supplementary Table S3**). After 10 days of shading treatment, the third leaf from the top of the trees was collected, quickly frozen in liquid nitrogen, and stored at -80°C for subsequent RNA-seq, gene cloning, and qRT-PCR experiments. The chlorophyll content of the leaf was determined immediately after collection using the method described by Han et al (Han et al., 2023).

### 2.6 Transcriptome sequencing and data analysis

Total RNA was extracted from young leaves using the Plant RNA Extraction Kit (Takara, Beijing, China). RNA purity and concentration were assessed with a NanoPhotometer^®^ spectrophotometer (IMPLEN, CA, USA), and the pure RNA was reverse-transcribed into cDNA using the PrimeScriptTM RT reagent Kit with gDNA Eraser (Takara, Beijing, China). For 12 samples (across shading intensities of 0%, 50%, 75%, and 95%, each with three replicates), cDNA libraries were prepared using the NEBNext^®^ Ultra™ RNA Library Prep Kit (Illumina, San Diego, CA, USA) and sequenced on an Illumina platform (NovaSeq 6000 sequencing system) by AZENTA (Beijing, China). Clean reads were aligned to the ‘N11-1’ chestnut genome using the Hisat2 v2.0.5 software. Gene expression levels were quantified with FeatureCounts tool (http://subread.sourceforge.net/), and FPKM (Fragments Per Kilobase pair per Million read) values were calculated based on transcript lengthes. Differentially expressed genes (DEGs) were identified using the DESeq2 software (http://bioconductor.org/packages/release/bioc/html/DESeq2.html) with |log2FC| > 1 and P_adj_ < 0.05 as thresholds. Heatmaps generated with TBtools visualized DEG expression patterns across treatments (Chen et al., 2023a). For further analysis, a trend analysis of the common DEGs was conducted using the Gene Denovo online tool (https://www.omicshare.com/tools/Home/Soft/trend) (Mu et al., 2024) with a grouping threshold of 20 and a significance level <0.05. Only groups exceeding the threshold were considered valid (Wang et al., 2012). The Fimo: Binding Motif Scan module in TBtools was used to identify upstream transcription factors associated with *CmLhcb2.1*, leveraging the PlantTFDB database (http://plantregmap.gao-lab.org/) and the InterProScan database (https://www.ebi.ac.uk/interpro/result/interprosca/) (Chen et al., 2023a). Finally, OmicsSuite software was used to assess the correlations between *CmLhcb2.1* and the upstream transcription factors, and their relationships were visualized using Cytoscape v3.10.0 software (Miao et al., 2024).

### 2.7 Quantitative reverse transcription PCR

Total RNA obtained from 12 samples was used to perform qRT-PCR with the SYBR PrimeScript RT-PCR Kit (Takara, Beijing, China) on an ABI 7500 Real-Time PCR system (Thermofisher, ABI 7500, Singapore) using the primers designed with the Batch qPCR Primer Design feature in TBtools. The relative expression levels of the genes were calculated using the 2^-ΔΔCT^ method (Livak and Schmittgen, 2001) and chestnut Actin (*CmActin*) as the internal reference gene (Chen et al., 2023a, 2019). The information on the primers is provided in **Supplementary Table S4**.

### 2.8 Subcellular localization

The first-strand cDNA was synthesized using the PrimeScriptTM RT reagent Kit with a gDNA Eraser from the total RNA (Takara, Beijing, China). Meanwhile, primers for gene cloning were designed using the Primer6 software (Koressaar and Remm, 2007) (**Supplementary Table S5**). Then, the open reading frames (ORFs) of *CmLhcb2.1* and *CmGLK* were amplified using the designed primers, employing TaKaRa LA Taq^®^ with GC Buffer (Takara, Beijing, China). The ORF sequences of *CmLhcb2.1* and *CmGLK* genes lacking the stop codon were ligated to the pAN580 vector using seamless cloning technology, and GFP was fused to the N-terminus. These recombinant vectors were introduced into the *Arabidopsis thaliana* protoplasts via polyethylene glycol (PEG)-mediated transformation. The GFP fluorescence signals were finally visualized using a laser confocal microscope (470 nm excitation light) to analyze the subcellular localization of CmLhcb2.1 and CmGLK proteins within the cells (Yoo et al., 2007).

### 2.9 Overexpression of *CmLhcb2.1* in tobacco

The ORF sequence of the *CmLhcb2.1* gene was ligated into the pBWA(V)HS vector containing the CaMV35S promoter using the seamless cloning method. The recombinant plasmid was then transformed into *Agrobacterium tumefaciens* GV3101 cells and used to infect the tissue cultured tobacco leaves using a infiltrating method (Mark et al., 1990). The positive transgenic tobacco plants were rooted and transplanted into sterile substrates, and then cultivated in a light-dark cycle of 18:6 in a growth chamber for 50 days. Then the seedlings were subjected to shading treatment using black shading nets, four shading intensities treatments (0%, 50%, 75%, and 95%) were adopted in this experiment. The content of chlorophyll in the leaves of transgenic and wild-type tobacco was measured using a UV spectrophotometer (Han et al., 2023). In addition, the malondialdehyde (MDA) content was measured following an Enzyme-Linked Immunosorbent Assay (ELISA) (Chang et al., 2024).

### 2.10 Yeast one-hybrid (Y1H) assay and dual-luciferase reporter assay (LUC)

The ORF sequence of the *CmGLK* gene was ligated into the pGADT7 effector vector. Meanwhile, the 2000 bp promoter sequence upstream of the ATG start codon of the *CmLhcb2.1* gene was amplified by PCR using the primers listed in **Supplementary Table S5** and inserted into the pAbAi vector to construct the reporter vector. The auto-activation test revealed that 100 ng/mL of Aureobasidin A (AbA) could effectively inhibit yeast growth. Finally, both the recombinant vectors were transformed into the YIH Gold cells, and their interaction was assessed based on yeast growth on an Synthetic Dropout - Uracil and Leucine (SD-UL) selection medium supplemented with 100 ng/mL AbA.

The ORF of the *CmGLK* gene was ligated into the pGreen II 62-SK effector vector, while the 2000 bp promoter sequence of the *CmLhcb2.1* gene was ligated in front of the LUC gene in the pGreen II 0800-LUC vector. The obtained recombinant vectors were introduced into the *Agrobacterium tumefaciens* GV3101 cells and used to infect tobacco leaves using a injecting method (Mark et al., 1990). After infection, the tobacco plants were cultured in the dark for 24 hours and transferred to a growth chamber at 25°C with an alternating cycle of 16 hours of light and 8 hours of darkness for two days. The leaves were further analyzed for the LUC fluorescence signal using a Chemiluminescence imaging system (Tanon Science & Technology, Tanon 5200, Shanghai, China), and the LUC and REN activities were measured using the Dual-Luciferase Reporter Assay Kit (Vazyme, DL101-01, Nanjing, China). Finally, the relative LUC/REN ratio was calculated.

### 2.11 Statistical analysis

The results of qRT-PCR, chlorophyll content, MDA content and photosynthetic parameters were subjected to variance analysis with Excel 2010 and SPSS 22 software. These data were represented as bar or line charts. GraphPad Prism 8 was used to plot the bar and line charts.

## 3 Results

### 3.1 Phylogenetic relationships of CmLhca/bs

**Figure 1:**
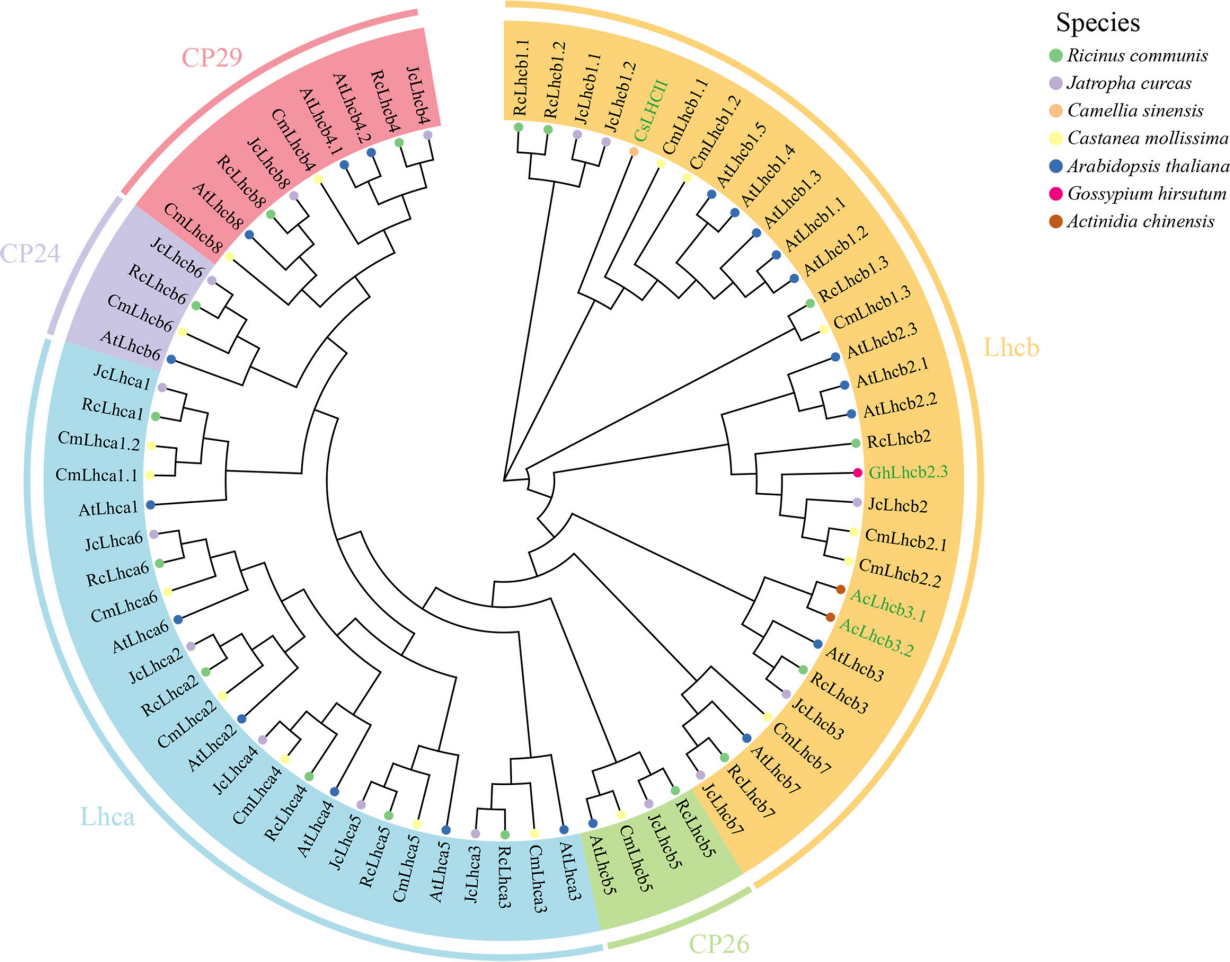
Phylogenetic analysis of the Lhca/b proteins of Castanea mollissima, Arabidopsis thaliana, Ricinus communis, Jatropha curcas, Camellia sinensis, Gossypium hirsutum, and Actinidia chinensis. The FastTree software based on the maximum likelihood method was used to construct the phylogenetic tree. The members were clustered into five clades (Lhca, Lhcb, CP24, CP26, and CP29). In the figure, the prefix “At-” in the names represents a member of the A. thaliana Lhca/b family proteins, and “Rc-”, “Jc-”, “Cs-”, “Cm-”, “Gh-”, and “Ac-” represent members of the R. communis, J. curcas, C. sinensis, C. mollissima, G. hirsutum, and A. chinensis, respectively. Among the different proteins, GhLhcb2.3, AcLhcb3.1, AcLhcb3.2, and CsLHCII (shown in green font) have been associated with chlorophyll synthesis.

The present study identified 17 members of the CmLhca/b family from the ‘N11-1’ chestnut genome, as listed in **Table 1**. These members, along with the previously reported Lhca/b family proteins from Arabidopsis, castor bean, and *Jatropha curcas*, were used to construct a phylogenetic tree (**Figure 1**, **Supplementary Table S1**) (Zhao et al., 2020b) to assess the evolutionary relationship. The approach divided the CmLhca/b proteins into five subgroups: the Lhca, the Lhcb, the CP24, the CP26, and the CP29 groups (**Figure 1**). The Lhca group had seven family members, the Lhcb group had six, the CP24 and CP26 groups had one each, and the CP29 group had two. On the evolutionary tree, CsLHCII of tea was closely related to CmLhcb1.1 and CmLhcb1.2, while GhLhcb2.3 of upland cotton was closely related to CmLhcb2.1 and CmLhcb2.2, suggesting that CmLhcb1.1, CmLhcb1.2, CmLhcb2.1, and CmLhcb2.2 might be involved in chlorophyll synthesis in the Chinese chestnut similar to CsLHCII and GhLhcb2.3. All these genes were classified within the Lhcb subgroup, indicating their potentia shared role in chlorophyll synthesis across different species.

**Table 1:** Physicochemical properties and subcellular localization of the CmLhca/b proteins.

Gene ID	Protein Name	Number of Amino Acid	Molecular Weight (Da)	Theoretical pI	Grand Average of Hydropathicity	Subcellular Location
*EVM0001721*	CmLhca1.1	244	26320.17	6.22	-0.121	chloroplast thylakoid membrane
*EVM0019356*	CmLhca1.2	148	16293.95	8.57	0.076	chloroplast outer membrane
*EVM0005463*	CmLhca2	270	29109.43	7.72	0.018	chloroplast outer membrane
*EVM0027158*	CmLhca3	273	29488.65	6.31	-0.077	chloroplast outer membrane
*EVM0020364*	CmLhca4	252	27838.92	6.22	-0.105	chloroplast outer membrane
*EVM0025461*	CmLhca5	263	28703.43	8.4	0.162	mitochondrial membrane
*EVM0017583*	CmLhca6	261	29087.77	7.67	-0.067	mitochondrial membrane
*EVM0032709*	CmLhcb1.1	243	25778.55	4.68	0.196	organelle membrane
*EVM0012458*	CmLhcb1.2	256	27406.33	5.46	-0.038	organelle membrane
*EVM0026912*	CmLhcb1.3	267	28466.38	5.29	-0.046	chloroplast thylakoid membrane
*EVM0002044*	CmLhcb2.1	265	28603.58	5.69	-0.042	organelle membrane
*EVM0016156*	CmLhcb2.2	246	26452.07	6.52	-0.092	organelle membrane
*EVM0010652*	CmLhcb4	271	29703.75	5.55	-0.098	organelle membrane
*EVM0011290*	CmLhcb5	288	30791.37	5.72	0.002	chloroplast outer membrane
*EVM0020797*	CmLhcb6	312	33813.8	6.53	0.08	organelle membrane
*EVM0023674*	CmLhcb7	246	27414.94	5.97	0.06	organelle membrane
*EVM0007317*	CmLhcb8	278	30647.16	6.23	0.015	mitochondrial membrane

### 3.2 Chromosomal localization and physicochemical properties of the *CmLhca/b* genes

**Figure 2:**
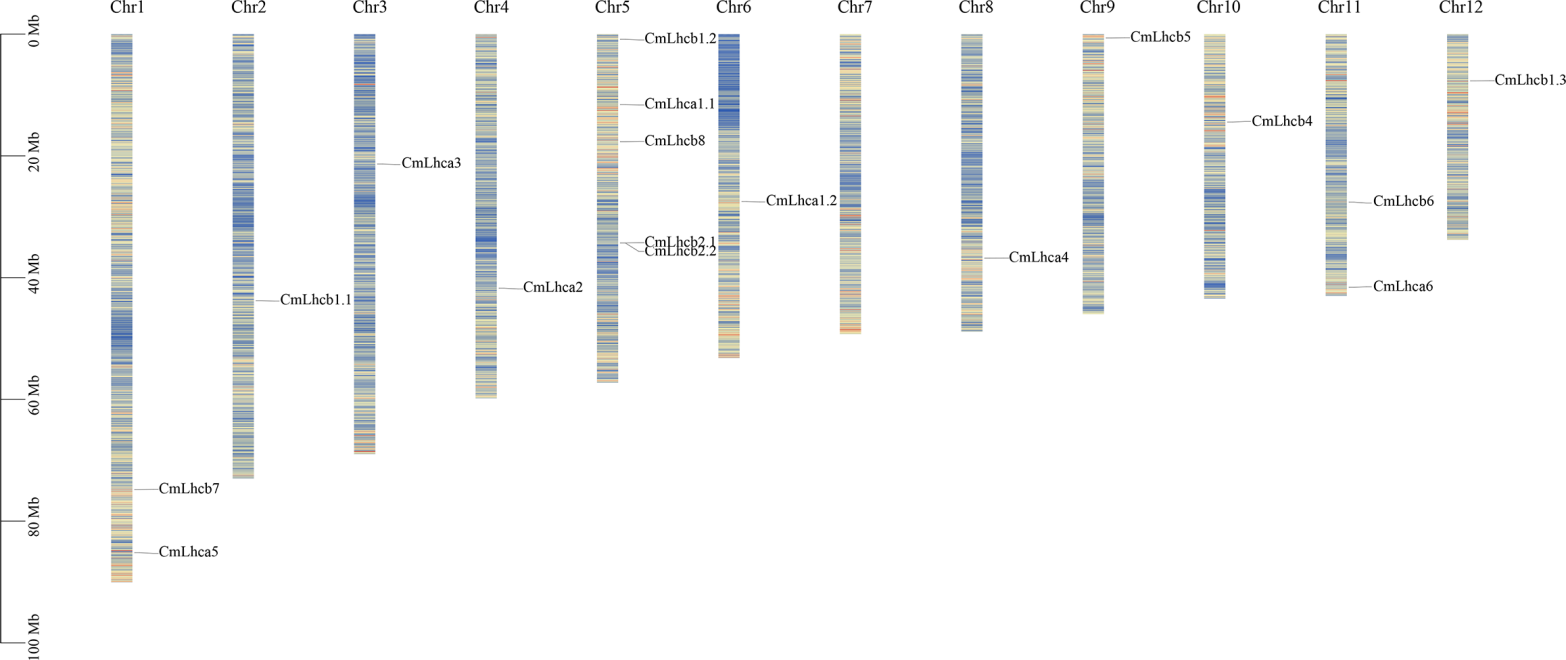
The distribution of the *CmLhca/b* genes on the Chinese chestnut chromosomes. Vertical colored bars represent the chromosomes of Chinese chestnut. The gene name and number are shown at the right/left of each chromosome. The scale bar on the left represents the length of the chromosomes.

Further analysis showed that the 17 *CmLhca/b* members were randomly distributed across 11 out of 12 chromosomes in Chinese chestnut. The *CmLhca/b* genes were most densely distributed on chromosome 5, with 5 members. Additionally, there are 2 members on chromosome 1 and chromosome 11, none on chromosome 7, and 1 member on each of the other chromosomes. (**Figure 2**).

Subsequent analysis of the physicochemical properties revealed that the number of amino acids in the CmLhca/b proteins ranged from 148 to 312, the molecular weight varied from 16,293.95 Da to 33,813.8 Da. The isoelectric points were between 4.68 and 8.57, among these, 13 out of 17 members were less than 7, indicating that these proteins had more acidic amino acids (Zhao et al., 2022, 2020). The grand average of hydropathicity (GRAVY) for all members was between -0.121 and 0.196, among these, 9 members had their GRAVYs less than 0 (**Table 1**), suggesting that these proteins were hydrophilic (Zou et al., 2020). Subcellular localization predicted that the 17 CmLhca/b proteins were on the membrane (**Table 1**). The protein sequences, lengths, positions, and other related information were listed in **Supplementary Table S2**.

### 3.3 Gene structure, protein motifs, and *cis*-acting elements of *CmLhca/b*s

**Figure 3:**
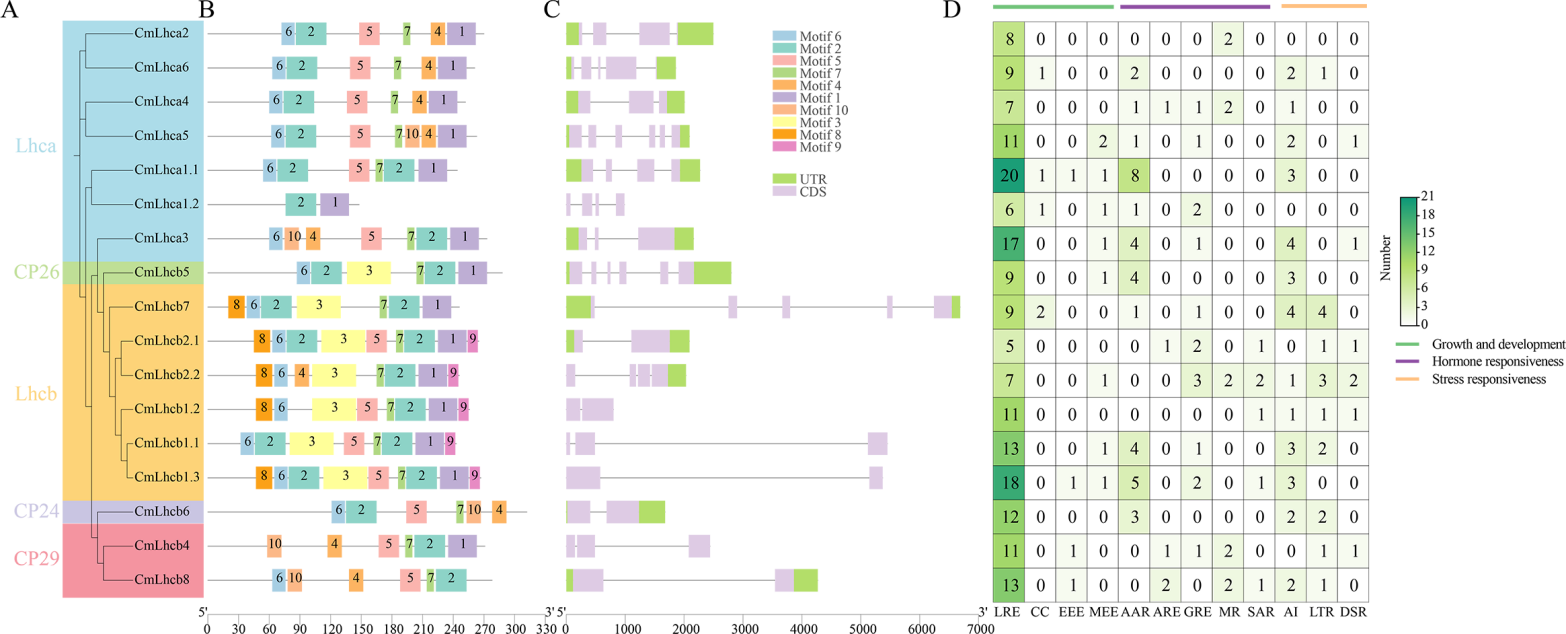
Motifs, gene structure, and *cis*-acting elements of *CmLhca/b* members of Chinese chestnut. **(A)** Phylogenetic tree based on the maximum likelihood method, **(B)** protein motif distribution, **(C)** gene structure, and **(D)** number of different types of *cis*-acting elements in the *CmLhca/b* promoters. The legend on the right side indicates the number and classification of various *cis*-elements. Here, LRE, CC, EEE, MEE, AAR, ARE, GRE, MR, SAR, AI, LTR and DSR represent the promoter for light responsive element, circadian control, endosperm expression element, meristem expression element, abscisic acid responsive element, auxin-responsive element, gibberellin-responsive element, methyl jasmonate responsiveness, salicylic acid responsiveness, anaerobic induction, low-temperature responsiveness and defense and stress responsiveness, respectively.

The study identified ten motifs conserved among the members of the *CmLhca/b* gene family which were named motif 1-10 (**Figures 3A, B**). The motifs within the members of different groups showed low consistency, motif 2, 5, 7 were present in all members, while motif 1 was present in all members except CmLhc6 and CmLhc8, and motif 6 existed all members except CmLhca1.2 and CmLhcb8. And certain motifs were unique to specific subgroups (**Figures 3A, B**). For instance, Motif 3 was present only in the Lhcb and CP26 subgroups, while Motif 8 and Motif 9 were found exclusively in the Lhcb subgroup. Motifs 1 to 3 corresponded to the different sequences of the Chlorophyll A-B binding domain (**Supplementary Table S6**) that might play different roles in photosynthesis.

Further analysis revealed significant differences among the *CmLhca/b* members in gene structure. The longest member, *CmLhcb7*, having the longest intron, was 6,688 bp long, while the shortest, *CmLhcb1.2*, was only 802 bp long. Additionally, 6 out of 7 members of the Lhca subgroup retained almost the entire UTR. The CP26 and CP24 also retained the entire UTR. Significant deletions were found in the UTR regions in most of the members of other subgroups, excluding *CmLhcb7* and *CmLhcb2.1*. The number of exons in the *CmLhca/b* genes ranged from one to six, introns from one to five, with only four members having more than three introns (**Figure 3C**), suggesting that *CmLhca/b* genes might perform more conserved functions (Liu et al., 2021).

*CmLhca/b* genes had promoters enriched with various *cis*-acting elements, mainly those related to growth and development, hormone response, and stress resistance (**Figure 3D**). Among these elements, The light-responsive elements were the most abundant in almost all members than other elements, suggesting that their primary function might be related to light reactions (Chen et al., 2023b). Elements related to hormone response were predominantly abscisic acid and gibberellin response elements, while elements related to stress resistance were mostly the anaerobic induction elements and low-temperature response elements. This suggested that *CmLhca/b* genes might be significantly influenced by these hormones and implies a potential role in modulating stress resistance and growth (Pietrzykowska et al., 2014).

### 3.4 Collinearity of the *CmLhca/b* genes within the Chinese chestnut genome and with other species

**Figure 4:**
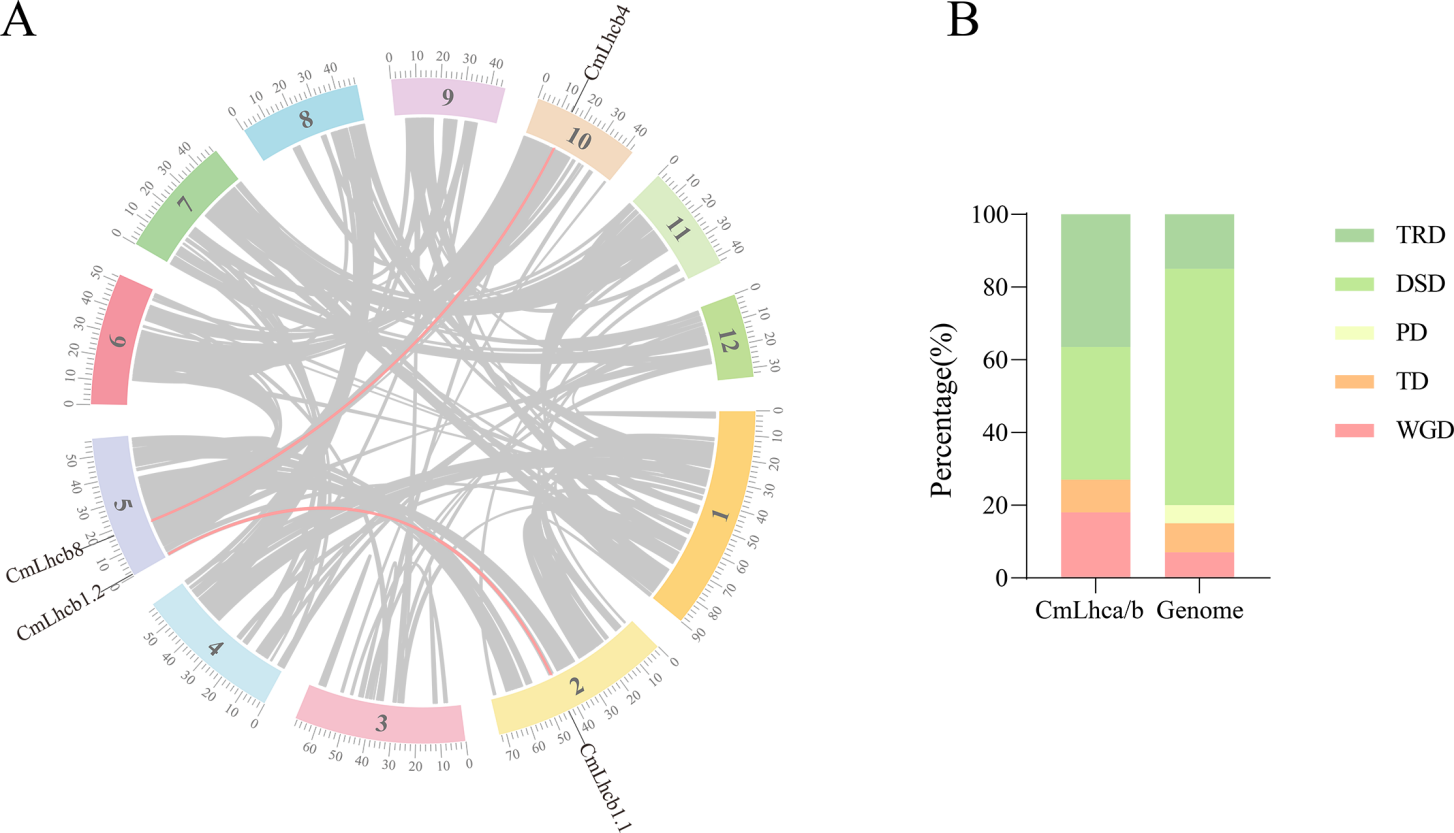
Analysis of collinearity and duplication types in the *CmLhca/b* gene family. **(A)** Intra-genomic collinearity of the *CmLhca/b* genes. **(B)** Duplication types of the *CmLhca/b* genes.

To further explore the duplication and evolutionary history, we analyzed the collinearity of the *CmLhca/b* genes within the Chinese chestnut genome and with other species using the MCScanX software. We identified two pairs of collinear genes, *CmLhcb1.1*/*CmLhcb1.2* and *CmLhcb4*/*CmLhcb8*, in the Chinese chestnut genome (**Figure 4A**). In-depth analysis revealed that the members of the *CmLhca/b* gene family had experienced four types of duplication events, namely TRD, DSD, TD, and WGD. This observation suggested that the PD type of the *CmLhca/b* genes was lost entirely from the chestnut genome, while the TRD and WGD duplication types have increased (**Figure 4B**). Additionally, among the 11 pairs of genes with different duplication types, five pairs yielded Ka/Ks values, which were less than 1 (**Table 2**), suggesting that they were subjected to purifying selection during evolution. Another six pairs’ Ka/Ks values showed that they were highly divergent gene sequences (**Table 2**), suggesting that these genes might have undergone positive selection or adaptive evolution, leading to significant changes in their sequences.

**Table 2:** Ka/Ks values of the duplicated gene pairs of the *CmLhca/b* family.

Gene pairs		Duplication types	Ka	Ks	Ka/Ks
*CmLhcb4*	*CmLhcb8*	WGD	0.217615696	0.929395679	0.234147523
*CmLhcb1.1*	*CmLhcb1.2*	WGD	0.048748119	0.857404248	0.056855467
*CmLhcb2.1*	*CmLhcb2.2*	TD	0.042981516	(NaN) Highly divergent gene sequences	
*CmLhcb5*	*CmLhcb7*	DSD	0.500609496	(NaN) Highly divergent gene sequences	
*CmLhca4*	*CmLhcb7*	DSD	0.592014462	(NaN) Highly divergent gene sequences	
*CmLhca4*	*CmLhcb6*	DSD	0.626661902	(NaN) Highly divergent gene sequences	
*CmLhcb6*	*CmLhcb7*	DSD	0.654745229	(NaN) Highly divergent gene sequences	
*CmLhca2*	*CmLhca6*	TRD	0.35227854	1.567391334	0.224754682
*CmLhca1.1*	*CmLhca1.2*	TRD	0.123975713	0.189017736	0.655894602
*CmLhcb1.2*	*CmLhcb1.3*	TRD	0.087242865	3.70085545	0.023573702
*CmLhca3*	*CmLhca5*	TRD	0.524523377	(NaN) Highly divergent gene sequences	

**Figure 5:**
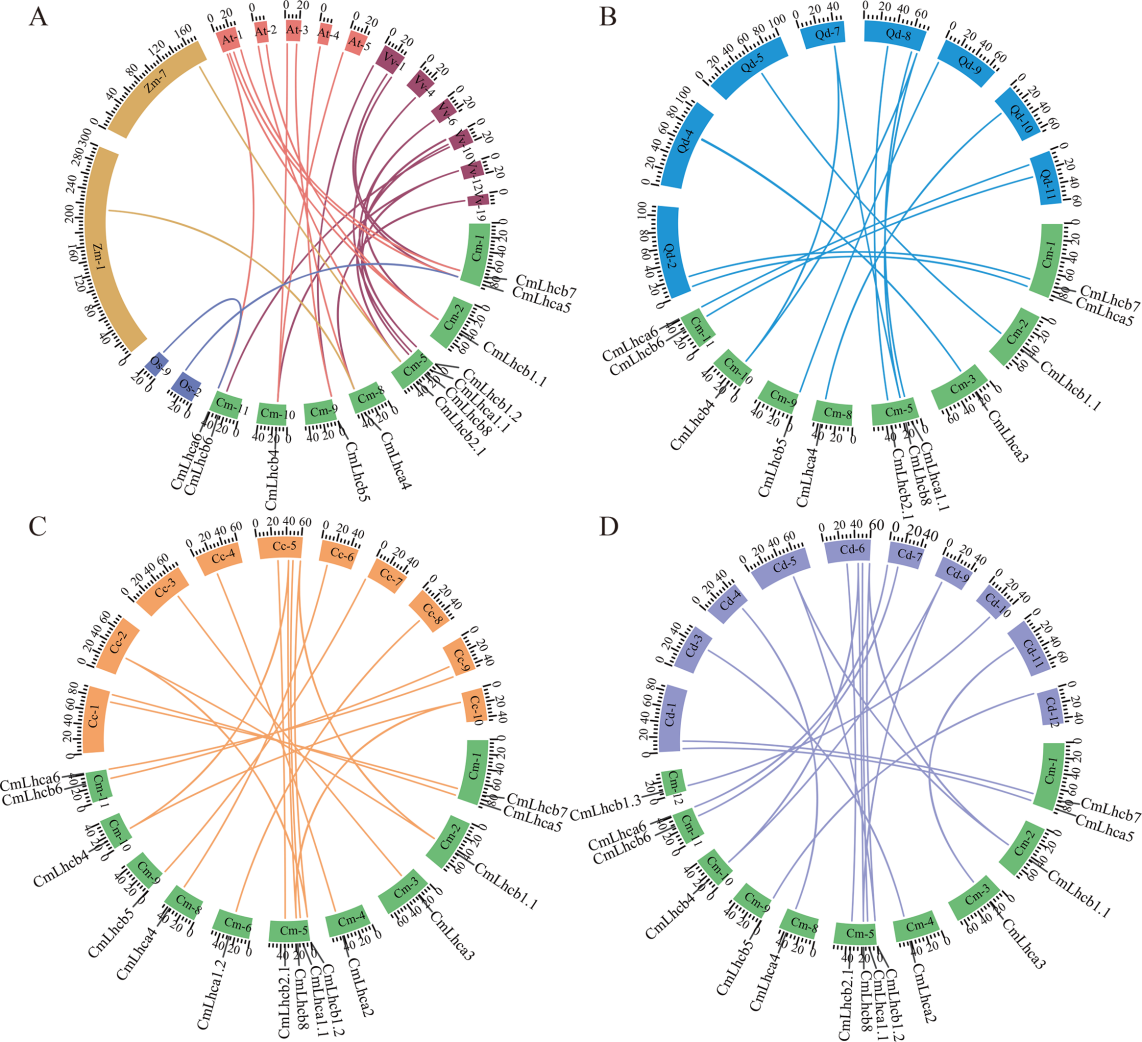
Collinearity analysis of the chestnut *CmLhca/b* genes with rice, maize, Arabidopsis, grape, oak, Japanese chestnut, and American chestnut. **(A)** Collinearity of the *CmLhca/b* gene family with rice, maize, Arabidopsis, and grape. Here, Cm-, Os-, Zm-, At-, and Vv- represent the chestnut, rice, maize, Arabidopsis, and grape chromosomes, respectively. **(B)** Collinearity of the *CmLhca/b* gene family with oak. Here, Qd- represents the oak chromosome. **(C)** Collinearity of the *CmLhca/b* gene family with Japanese chestnut. Here, Cc- represents the Japanese chestnut chromosome. **(D)** Collinearity of the *CmLhca/b* gene family with American chestnut. Here, Cd- represents the American chestnut chromosome.The number following the species code (Cm-, Os-, Zm-, At-, Vv-, Qd-, Cc- and Cd-) represents the chromosome number, and the scale numbers on each chromosome indicate the chromosome length. The gene names outside the ring are the chestnut gene names that are linearly paired with those of other species.

Furthermore, two members of the *CmLhca/b* gene family showed collinearity with rice, two members with maize, ten members with Arabidopsis, and ten members with grape (**Figure 5A**). In addition, 12 Chinese chestnut members showed collinearity with oak (**Figure 5B**), 15 with Japanese chestnut (**Figure 5C**), and 15 with American chestnut (**Figure 5D**). These observations suggested that most *CmLhca/b* genes were formed after the divergence of monocots and dicots and established before the divergence of *Fagaceae* species.

### 3.5 Leaf chlorophyll content and *CmLhca/b* expression in chestnut under low-light stress

**Figure 6:**
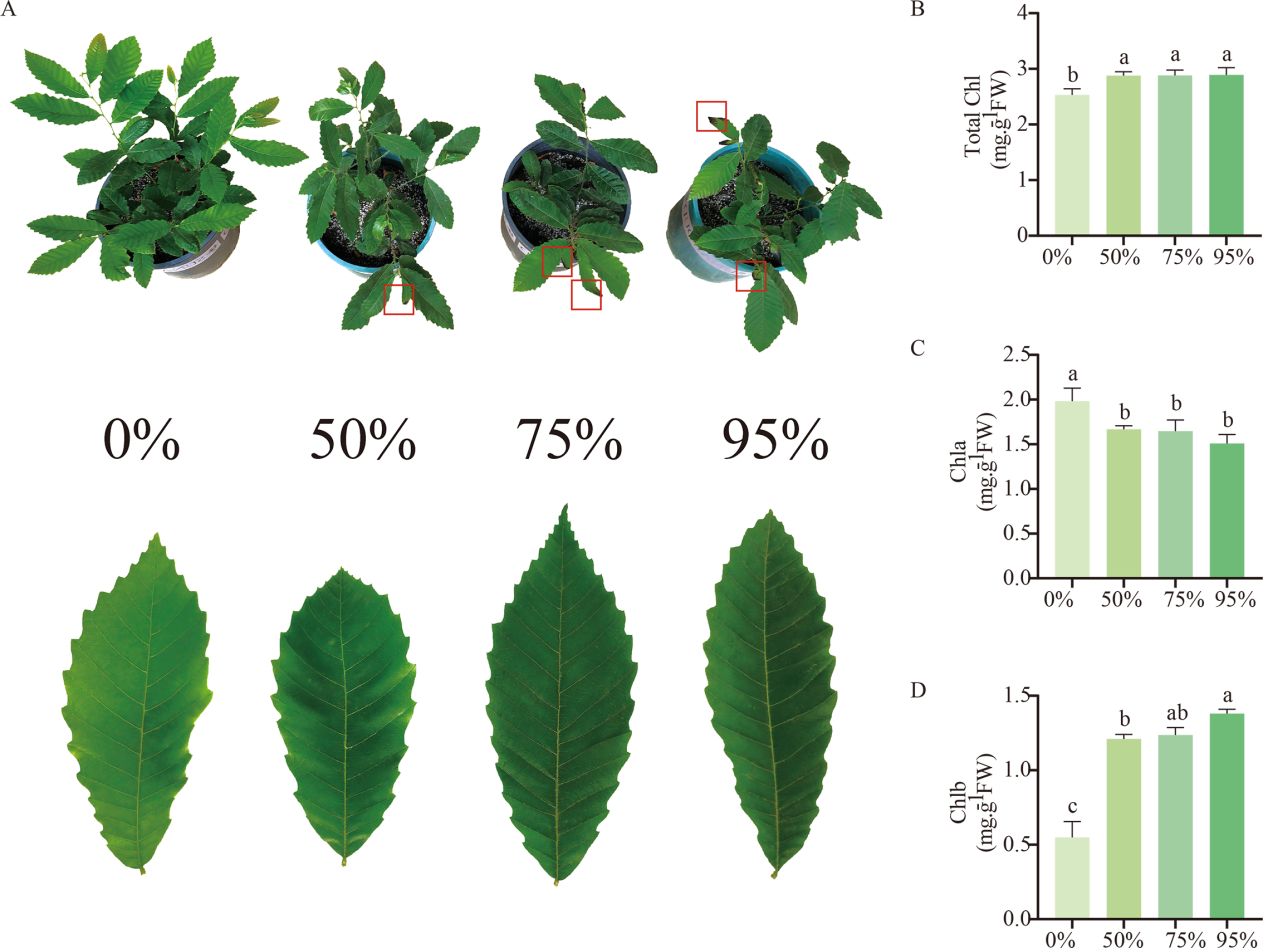
Changes in chestnut phenotype and leaf chlorophyll content after 10 days of exposure to shading treatment. **(A)** Development of chestnut seedlings and leaves after 10 days of shading treatment. The red boxes indicate the wilting and death of leaf margins due to low-light stress. **(B)** Chlorophyll content of leaves under different shading treatments. **(C)** Chlorophyll a content of leaves under different shading treatments. **(D)** Chlorophyll b content of leaves under different shading treatments. The letters above the bars indicate significant differences, derived from the T-test.

Furthermore, we subjected chestnut seedlings to shading treatment to investigate the expression characteristics of the *CmLhca/b* genes under low-light stress. By the 10^th^ day of shading treatment, the leaves were damaged; the degree of leaf damage intensified with the increase in shading intensity (**Figure 6A**). Besides, the leaf color became darker with the rise in shading intensity (**Figure 6A**). Further analysis revealed significant differences in leaf chlorophyll content among the different shading intensities (**Figure 6B**). The total chlorophyll content significantly increased after shading treatment compared with the control, while the chlorophyll a content significantly decreased. However, no significant differences were observed in these two parameters among the different shading intensities (**Figures 6B, C**). Interestingly, the chlorophyll b content in the leaves gradually increased with an increase in shading intensity (**Figure 6D**).

**Figure 7:**
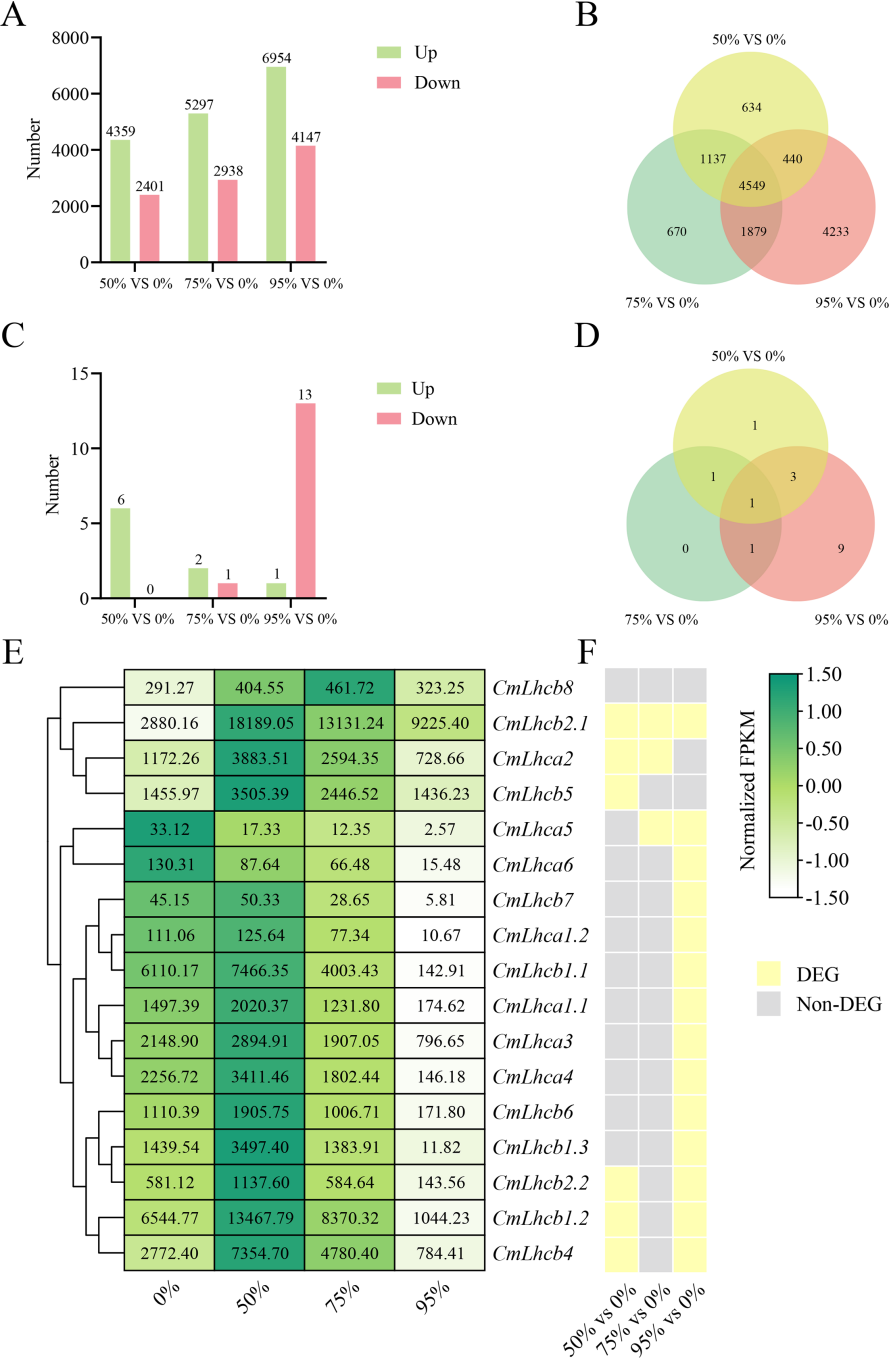
Analysis of the differentially expressed genes of Chinese chestnut under different shading treatments. **(A)** The number of differentially expressed genes (DEGs) in chestnut under different shading intensities. Green bars represent upregulated genes, and pink bars represent downregulated genes. **(B)** Venn diagram shows the unique and shared DEGs under different shading intensities. **(C)** The number of differentially expressed *CmLhca/b* genes under different shading intensities. **(D)** Venn diagram shows the unique and shared differentially expressed *CmLhca/b* genes under different shading intensities. **(E)** FPKM values of *CmLhca/b* genes under different shading intensities. **(F)** Differential expression of *CmLhca/b* genes under different shading intensities. Yellow indicates differential expression, and grey indicates non-differential expression.

Subsequent transcriptome sequencing and data analysis revealed that under 50% shading intensity, 6,760 genes were differentially expressed compared to the non-shaded group, including 4,359 upregulated and 2,401 downregulated genes. Under 75% shading, the number of DEGs increased to 8,235, with 5,297 upregulated and 2,938 downregulated genes. Under 95% shading intensity, the number of DEGs was 11,101, including 6,954 upregulated and 4,147 downregulated genes (**Figure 7A**). A total of 4,549 common genes were differentially expressed under all three shading intensities compared with the non-shaded control (**Figure 7B**). Among these, six *CmLhca/b* gene family members were differentially expressed at 50% shading intensity, three at 75% shading intensity, and fourteen at 95% shading intensity (**Figure 7C**). Notably, only one member *CmLhcb2.1* showed commonly differential expression under all three shading intensities (**Figure 7D**). Further analysis of the expression changes in the *CmLhca/b* gene family members revealed that the only *CmLhcb2.1* was upregulated under all shading intensities (**Figures 7D–F**).

### 3.6 Role of *CmLhcb2.1* in regulating leaf chlorophyll content and resistance of tobacco under low-light stress

**Figure 8:**
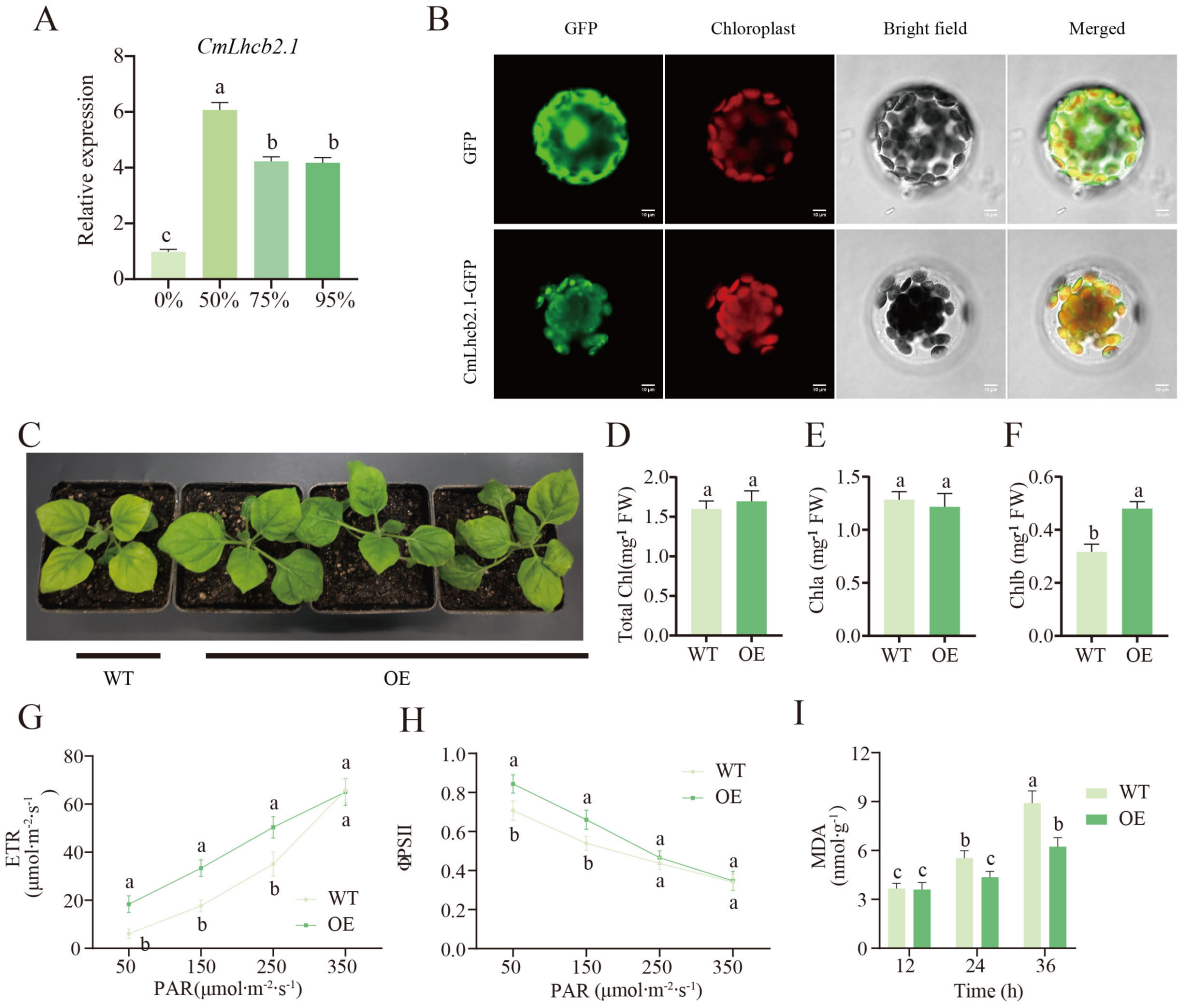
Functional characterization of the *CmLhcb2.1* gene. **(A)** Relative expression level of *CmLhcb2.1* under different shading intensities. **(B)** Subcellular localization of the *CmLhcb2.1* gene. **(C)** The phenotype of tobacco after *CmLhcb2.1* overexpression. The tobacco leaves overexpressing *CmLhcb2.1* (OE) are greener than the wild type (WT). **(D–F)** Differences of total chlorophyll, chlorophyll a, and chlorophyll b content in *CmLhcb2.1-*overexpressing and the wild type lines. OE and WT represent *CmLhcb2.1-*overexpressing and the wild type lines respectively, as shown in the image below. **(G, H)** Differences in electron transport rate (*ETR*) and photochemical quantum efficiency (*φPSII*) between OE and WT lines under different shading conditions. **(I)** Differences in malondialdehyde (MDA) content between OE WT lines. The letters above the bars and points indicate significant differences, as determined by the T-test.

Further qRT-PCR analysis confirmed that the expression levels of *CmLhcb2.1* in chestnut leaves after 10 days of exposure to different shading treatments were all significantly higher than that under the non-shading condition, among them, the expression level was highest at 50% shading treatment (**Figure 8A**).This result suggested that the *CmLhcb2.1* might play a key role in the Chinese chestnut’s response to low-light stress. Subcellular localization showed that the fluorescence signal of the CmLhcb2.1-GFP fusion protein largely overlapped with the fluorescence signal of the chloroplast marker, confirming that the CmLhcb2.1 protein is located in the chloroplasts (**Figure 8B**).

Our initial experiments showed that the *CmLhcb2.1* expression level and the chlorophyll b content were significantly high under low-light stress (**Figure 8D**). To further investigate whether *CmLhcb2.1* is a key gene regulating chlorophyll b synthesis under low-light stress, the gene was successfully transformed into tobacco to generate the OE lines (OE). The leaves of the OE lines significantly turned greener than the wild line (WT) (**Figure 8C**). A significant increase in chlorophyll b content was found in the leaves of the OE line compared to the wild line (**Figure 8F**), while the total chlorophyll and chlorophyll a showed no significant changes (**Figures 8D, E**). More interestingly, under low light conditions, the growth of the OE line tobacco was significantly better than that of the wild type (**Figure 8C**), indicating greater tolerance to low-light stress. Additionally, under low light (50–95% shading intensities), the electron transport rate (*ETR*) and photochemical quantum efficiency (*φPSII*) of the OE line were significantly higher than those of the WT line (**Figures 8G, H**), indicating higher photosynthetic efficiency of the OE line under low light. Meanwhile, after exposure to 95% shading intensity (low light) for 24–36 hours, the MDA content in the WT line was significantly higher than that in the OE line (**Figure 8I**), suggesting that the cells or tissues of the WT line were more severely damaged. These observations indicated that tobacco overexpressing *CmLhcb2.1* had an increased chlorophyll b content and a higher resistance to low-light stress compared to the wild-type line. This suggested that *CmLhcb2.1* might enhance resistance to low-light stress by promoting the synthesis of chlorophyll b.

### 3.7 Analysis of transcription factors regulating *CmLhcb2.1*

**Figure 9:**
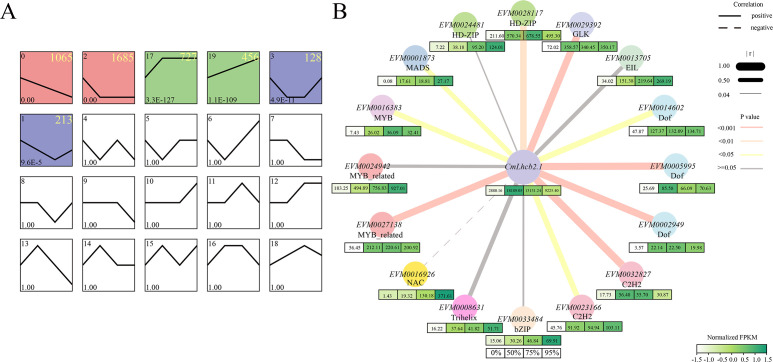
Trend analysis of differentially expressed genes under shading and analysis of co-expression genes interacting with *CmLhcb2.1*. **(A)** Trend analysis of 4,549 differentially expressed genes under shading conditions. The differentially expressed genes were divided into six groups: group 0, group 2, group 17, group 19, group 3, and group 1. Here, group 0 and group 2 represent the downregulated groups, group 17 and group 19 represent the upregulated groups, and group 3 and group 1 show downregulation followed by upregulation. The colored boxes indicate different trends groups. The yellow numbers in the top right corner of each box represent the number of genes of each trend. **(B)** Co-expression genes of *CmLhcb2.1* and their expression levels under different shading intensities. The thickness of box’s color below each gene represents the gene’ FPKM values related to expression levels under different shading intensities (0%, 50%, 75%, and 95%). The line type and thickness represent the correlation between *CmLhcb2.1* and its regulatory factors. Here, r represents the Pearson correlation coefficient, and the P-value tested with FPKM value represents significance.

To delve into the regulatory mechanism of *CmLhcb2.1*, we conducted trend analysis on 4,549 common DEGs identified under 50%, 75%, and 95% shading intensities. A total of 4,274 genes were clustered into 6 groups: group 0, group 2, group 17, group 19, group 3, and group 1, among which group 0 and group 2, containing a total of 2,750 genes, represented the downregulated group, group 17 and 19, containing a total of 1,183 genes, represented the upregulated groups; and group 3 and group 1, containing a total of 341 genes, showed downregulation followed by upregulation (**Figure 9A**). The *CmLhcb2.1* was found in the upregulated group 17. Therefore, we conducted an analysis of the co-expression transcription factors of *CmLhcb2.1* within the upregulated groups that might positively regulate its expression. Based on this approach, we identified 16 potential transcription factors that might interacted with *CmLhcb2.1.* (**Figure 9B**). Subsequent FPKM analysis of these 16 transcription factors revealed that only 10 transcription factors were significantly positively correlated with *CmLhcb2.1* expression (P < 0.05; **Figure 9B**). These 10 genes included two C2H2 zinc finger proteins, three Dof family members, one GLK transcription factor, one HD-ZIP family member, one MADS nuclear protein, one MYB family member, and one MYB-related protein.

**Figure 10:**
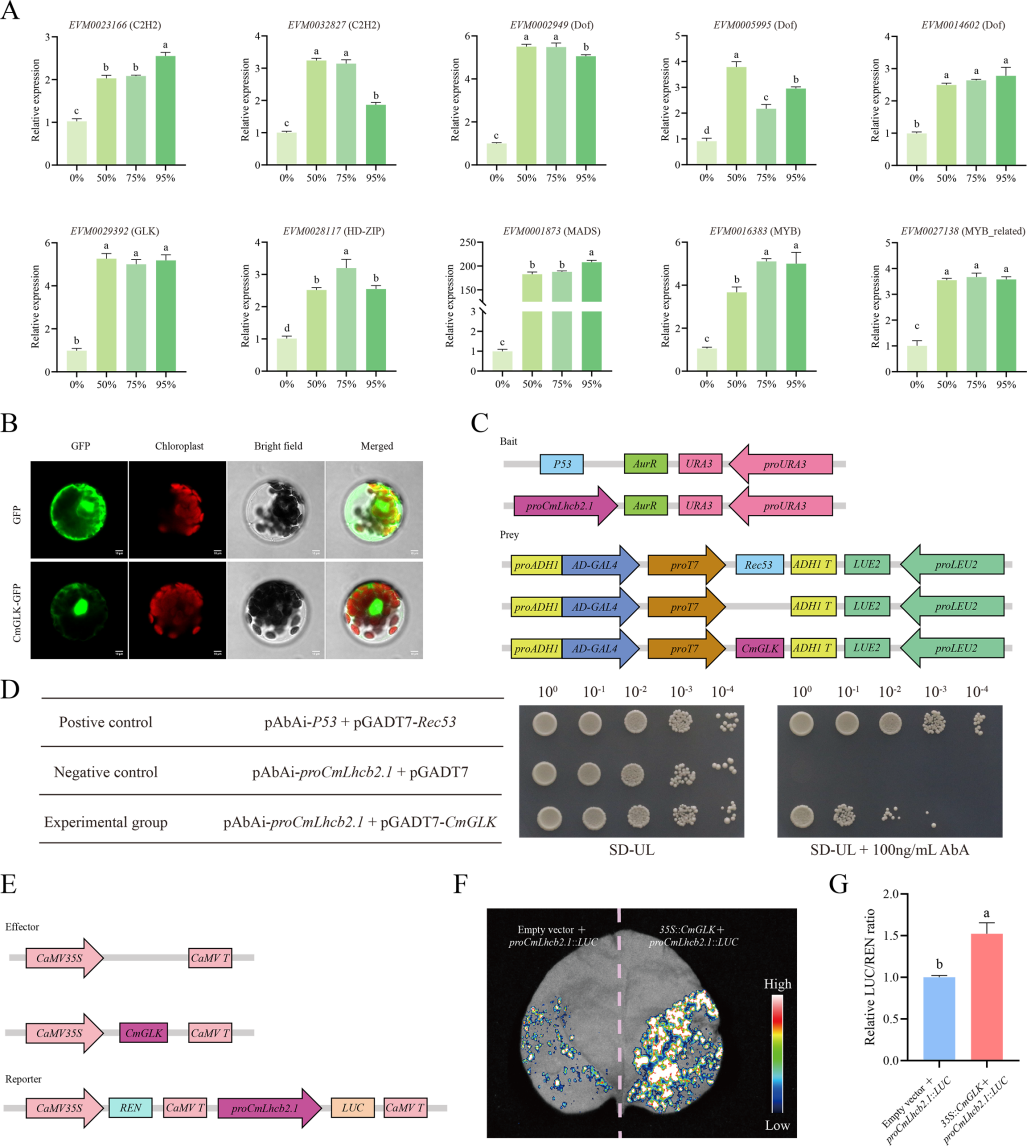
Interaction between *CmGLK* and *CmLhcb2.1*. **(A)** Relative expression levels of 10 co-expressed genes with *CmLhcb2.1* under different shading conditions in ‘Yanbao’ chestnut leaves. **(B)** Subcellular localization of *CmGLK* transcription factor. **(C)** Constructs used in the Y1H assay. **(D)** Growth of yeast transformed with different vectors. Growth of yeast cells carrying both *proCmLhcb2.1*-AbAi and pGADT7-*CmGLK* vectors indicates activation of the reporter gene. Here, 10^0^, 10^-1^, 10^-2^, 10^-3^, and 10^-4^ represent the dilution factors of the yeast solution used in the assay, and SD-UL represents SD medium lacking uracil (URA) and leucine (LEU). **(E)** Constructs used in the LUC assay. **(F)** Intensity of luciferase activity in leaves transformed with *35S::CmGLK* and *proCmLhcb2.1::LUC*. Stronger luciferase activity signals indicate greater promotion of *CmLhcb2.1’*s expression by the transcription factor. **(G)** Relative LUC/REN fluorescence intensity. The letters above the bars and points indicate significant differences, as determined by the T-test.

Further, qRT-PCR revealed that the 10 transcription factor genes were significantly upregulated under all shading intensities compared to the non-shaded group (**Figure 10A**). Based on these results and previous studies, the *CmGLK*, likely interacted with *CmLhcb2.1* that was involving in chloroplast development, was selected to verify its interaction with *CmLhcb2.1*. Subcellular localization revealed that the fluorescence signal due to the CmGLK-GFP fusion protein was separated entirely from the chloroplast fluorescence signal, and a strong fluorescence signal was observed in the nuclear region, demonstrating that the CmGLK protein is localized in the nucleus (**Figure 10B**). This indicated that *CmGLK* might regulate the expression of *CmLhcb2.1* as a transcription factor.

Further, the effector gene (*CmGLK*) and reporter vector (*CmLhcb2.1*) were constructed (**Figure 10C**) and transformed into Y1H Gold yeast strains to verify the interaction between the two. When the *proCmLhcb2.1*-AbAi and pGADT7-*CmGLK* vectors were co-transformed, the yeast cells grew on screening synthetic dropout medium lacking uracil and leucine (SD-UL) containing 100 ng/mL AbA (**Figure 10D**), indicating that the transcription factor *CmGLK* could specifically bind to *proCmLhcb2.1*.

A LUC assay further validated the Y1H assay. The effector gene (*CmGLK*) and reporter vector (*CmLhcb2.1*) for the LUC assay were constructed (**Figure 10E**) for use in tobacco. The tobacco leaves co-transfected with *35S::CmGLK* and *proCmLhcb2.1::LUC* showed significantly higher luciferase activity than the leaves transfected with *proCmLhcb2.1::LUC* alone (**Figure 10F**), suggesting *CmGLK* could specifically bind to *proCmLhcb2.1* and promoted the expression of it. Quantitative analysis confirmed that the leaves co-transfected with *35S::CmGLK* and *proCmLhcb2.1::LUC* had more luciferase activity than the leaves transfected with *proCmLhcb2.1::LUC* alone, indicating that *CmGLK* could specifically bind to *proCmLhcb2.1* and exert a positive regulatory effect (**Figure 10G**).

## 4 Discussion

Plants have evolved multiple strategies to adapt to low-light stress, including the regulation of gene expression (Wang et al., 2022). Recent studies have proved the *Lhca/b* gene family plays an important role in responding to low-light stress. However, research on chestnuts is limited (Li et al., 2024, 2022; Zhang et al., 2022). Notably, chestnut is grown in mountainous regions where low-light stress frequently occurs and faces yield challenges, demanding the need to investigate the intrinsic mechanisms of chestnut’s resistance to light stress, to identify and validate genes that confer resistance to low light stress, and to provide a foundation for targeted breeding of chestnuts to withstand light stress. Therefore, this study analyzed the entire chestnut genome and identified 17 *CmLhca/b* gene family members and their expression patterns under low-light stress. The phylogenetic tree was constructed including the 17 members of the *CmLhca/b* gene family and some *Lhca/b* genes of other species, among these, *GhLhcb2.3* in upland cotton and *CsLHCII* in tea have been confirmed to regulate the chlorophyll content of leaves (Chen et al., 2022; Zhang et al., 2021). Additionally chestnut *Lhcb* members *CmLhcb1.1* and *CmLhcb1.2* were grouped in the same clade as *CsLHCII*, while *CmLhcb2.1* and *CmLhcb2.2* were grouped in the same clade as *GhLhcb2.3*. Members on the same branch of the phylogenetic tree were often highly homologous to these known genes (Chen et al., 2022; Luo et al., 2022; Zhang et al., 2021). So this observation suggests that *CmLhcb1.1*, *CmLhcb1.2*, and *CmLhcb2.2* might also regulate chlorophyll content in the chestnut.

The Chlorophyll a/b binding domain is responsible for binding to chlorophyll molecules, forming photosynthetic complexes. These complexes can effectively absorb light energy, particularly in the blue and red regions of the visible spectrum (Chen et al., 2022). The binding domain is not only responsible for light absorption but also participates in transferring the absorbed light energy to the reaction center, facilitating the energy conversion process in photosynthesis. This process is the first step in energy transformation during photosynthesis, ultimately leading to the production of ATP and NADPH (Bryant and Gisriel, 2024; Li and Lin, 2024). The structural features of the Chlorophyll a/b binding domain help maintain the stability of the photosynthetic complexes. By binding with chlorophyll, these domains ensure the correct folding and function of the photosynthetic protein complexes. The Chlorophyll a/b binding domains may have different sequences and structures, allowing them to adapt to varying light conditions and environmental changes (Rankelytė et al., 2024). This adaptability may affect the photosynthetic efficiency and growth of plants. Certain Chlorophyll a/b binding domains may play a role in regulating photosynthesis, for example, by adjusting the rate of photosynthetic reactions in response to changes in light intensity to avoid photodamage (Zhang et al., 2023a). We found that the *CmLhca/b* gene family possesses three types (motif 1-3) of Chlorophyll a/b binding domain sequences. Besides, motif 2 was in all CmLhca/b proteins, motif 1 was in all except CmLhcb6 and CmLhcb8, and motif 3 was found only in the Lhcb and CP26 groups (So, CmLhca/b proteins including CmLhcb5, CmLhcb7, CmLhcb2.1, CmLhcb2.2, CmLhcb1.2, CmLhcb1.1 and CmLhcb1.3 in Lhcb and CP26 groups possesses the three types of Chlorophyll a/b binding domain). This observation indicates that Motif 3 is a unique Chlorophyll a/b binding structure of the *CmLhcb* genes, and the presence of this motif in the *CmLhcb* genes of these two groups suggests their special functions, probably associated with chlorophyll synthesis. The specific mechanisms involved need to be further studied.

Gene duplication is a primary mechanism by which plants generate new genes with new biological functions. This mechanism is crucial for plant evolution and ability to adapt to the environment. This study first analyzed the collinearity and duplication types of the *CmLhca/b* members across the chestnut genome (Qu et al., 2023). This approach revealed no PD (segmental) type for the *CmLhca/b* gene family members; however, the proportion of TRD (tandem) and WGD (whole-genome duplication) types was high, a result that has not been mentioned in other studies. This observation indicated the role of TRD and WGD in amplifying the *CmLhca/b* gene family. Besides, 5 out of 11 gene pairs with different duplication types had a Ka/Ks value less than 1, suggesting that these gene pairs might have undergone purifying selection, meaning that the negative effects of mutations were selectively eliminated. In contrast, the other 6 gene pairs had a Ka/Ks value greater than 1, suggesting that these gene pairs experienced positive selection, meaning that beneficial mutations were retained. In summary, this indicated that the expansion of the CmLhca/b gene family might have been achieved through specific selective pressures and gene duplication mechanisms (Zhang et al., 2023b). Subsequent inter-species collinearity analysis revealed the origins of the genes (Fu et al., 2024; Zhang et al., 2023b). Among the various members of the CmLhca/b gene family identified in this study, two exhibited collinearity with the monocot plants rice and maize, respectively. Additionally, ten members showed collinearity with Arabidopsis (a dicot plant), ten with grape (another dicot plant), twelve with oak (a dicot plant belonging to the Fagaceae family), fifteen with Japanese chestnut (also a Fagaceae dicot), and fifteen with American chestnut (another Fagaceae dicot). These findings suggest that most *CmLhca/b* genes originated after the divergence of monocots and dicots, but before the differentiation of the Fagaceae species.

We further performed RNA-seq to explore the expression patterns of *CmLhca/b* genes in chestnut under different shading intensities. Most genes were upregulated at 50% shading intensity but downregulated as the shading intensity increased, which might be due to excessive shading. This observation is similar to the decrease in *CsLhcb* expression reported in tea leaves under darkness, probably due to the influence of the biological clock (Hu et al., 2024).These observations indicate that shading can significantly impact the expression of light-harvesting complex genes, suggesting a complex relationship between light availability and gene regulation in chestnut. Interestingly, the *CmLhcb2.1* gene showed consistent upregulation under different low light intensities, suggesting its role in Chinese chestnut response to low-light stress. Moreover, the leaves of the chestnut seedlings under low-light stress turned considerably darker, and the content of chlorophyll b increased significantly compared with the non-shaded control. Thus, combining this observation with the conclusion from the evolutionary tree analysis, we speculate that *CmLhcb2.1* might have promoted chlorophyll b synthesis in the Chinese chestnut under low light, enhancing its adaptability. Numerous studies have shown that under low-light stress, plants typically increase the content of chlorophyll b to adapt to the environment (Alridiwirsah et al., 2018; Dai et al., 2009). However, the association between the increase in chlorophyll b content under low-light stress and the members of the *Lhca/b* gene family has not been reported in chestnut. Therefore, we overexpressed *CmLhcb2.1* in tobacco leaves and analyzed the differences in phenotype, chlorophyll content, photosynthetic efficiency, and MDA content between the gene overexpressing and the wild-type tobacco. The experiments showed that chlorophyll b content in the transgenic tobacco increased, and its adaptability to low-light stress enhanced notably, confirming *CmLhcb2.1* played an important role in resisting low-light stress.

Research has confirmed that transcription factors play a crucial role in plant adaptation to low-light stress (Wu et al., 2020; Zhang et al., 2018). Therefore, we searched for transcription factors in the differentially expressed genes of chestnut leaves under shading and non-shading treatments that had a significant positive correlation with the expression level of CmLhcb2.1, and identified 10 transcription factors, including the transcription factor *CmGLK*. Subcellular localization revealed *CmGLK* in the nucleus, confirming its role as a transcription factor. Subsequent Y1H and LUC assays confirmed that *CmGLK* could bind to the *CmLhcb2.1* promoter and enhance its expression. These results indicated that under low-light stress, *CmGLK* positively regulated *CmLhcb2.1* to enhance chlorophyll b synthesis in Chinese chestnut, thereby improving its resilience to low-light stress. Numerous studies have demonstrated that *GLK* transcription factors influence chlorophyll content in plants by regulating *LHC* genes. For instance, the introduction of *ZmGLK* from maize into rice promoted leaf chlorophyll content (Yeh et al., 2022), and overexpression of *CaGLK2* in red pepper enhanced the chlorophyll content of green fruits (Brand et al., 2014). Additionally, studies have reported that *AtGLK* transcription factors regulate the expression of *AtLhca/b* genes in Arabidopsis and control chloroplast function (Waters et al., 2009). All these studies indicate that *GLK* transcription factors play an important role in regulating chlorophyll content in plants by modulating the expression of *LHC* genes, further emphasizing their critical role in plant photosynthesis and adaptability.

## 5 Conclusions

This study reveals the response mechanisms of Chinese chestnut under low light stress, particularly highlighting the importance of the *CmLhcb2.1* gene in enhancing photosynthetic capacity and resistance to low light conditions. Through the identification of 17 *CmLhca/b* gene members and their phylogenetic analysis, we found that *CmLhcb2.1* is closely related to homologous genes in other plants, indicating its critical role in photosynthetic adaptation. Experimental results demonstrated that the expression of *CmLhcb2.1* was significantly upregulated under low light conditions, further confirming its importance in the synthesis of photosynthetic pigments. Additionally, transgenic tobacco experiments showed that the overexpression of *CmLhcb2.1* effectively improved the plant’s tolerance to low light stress. Finally, the positive regulation of *CmLhcb2.1* by the transcription factor *CmGLK* further elucidates its role in light adaptation. These findings provide a theoretical basis for the targeted breeding of Chinese chestnut, aiming to enhance its yield and survival ability in low light environments. The function of *CmLhcb2.1* has not yet been verified in the chestnut species in this study, which will be our next focus of work.

## Data Availability

The clean transcriptome sequence data reported in this paper have been deposited in Genome Sequence Archive (GSA) in National Genomics Data Center (NGDC, https://ngdc.cncb.ac.cn/gsa), China National Center for Bioninformation/Beijing Institute of Genomics, Chinese Academy of Sciences (GSA: CRA022911).
